# The evolution of photosynthesis in chromist algae through serial endosymbioses

**DOI:** 10.1038/ncomms6764

**Published:** 2014-12-10

**Authors:** John W. Stiller, John Schreiber, Jipei Yue, Hui Guo, Qin Ding, Jinling Huang

**Affiliations:** 1 Department of Biology, East Carolina University, Greenville, North Carolina 27858, USA; 2 Department of Computer Science, East Carolina University, Greenville, North Carolina 27858, USA

## Abstract

Chromist algae include diverse photosynthetic organisms of great ecological and social importance. Despite vigorous research efforts, a clear understanding of how various chromists acquired photosynthetic organelles has been complicated by conflicting phylogenetic results, along with an undetermined number and pattern of endosymbioses, and the horizontal movement of genes that accompany them. We apply novel statistical approaches to assess impacts of endosymbiotic gene transfer on three principal chromist groups at the heart of long-standing controversies. Our results provide robust support for acquisitions of photosynthesis through serial endosymbioses, beginning with the adoption of a red alga by cryptophytes, then a cryptophyte by the ancestor of ochrophytes, and finally an ochrophyte by the ancestor of haptophytes. Resolution of how chromist algae are related through endosymbioses provides a framework for unravelling the further reticulate history of red algal-derived plastids, and for clarifying evolutionary processes that gave rise to eukaryotic photosynthetic diversity.

## Abstract (web summary)

The chromalveolate hypothesis proposes that chromist algae became photosynthetic through a single endosymbiosis in a common ancestor. Here, Stiller *et al*. use a novel statistical approach to propose that instead, the major chromist algae arose as a result of three specific serial plastid transfers.

## Graphical abstract

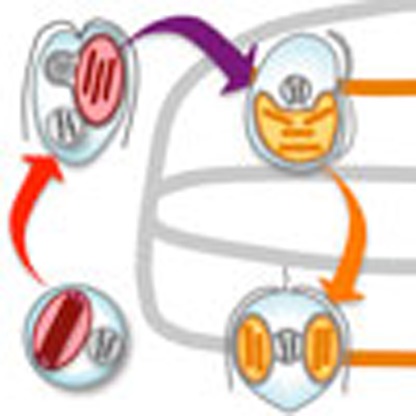

Photosynthesis moved into eukaryotic organisms through the endosymbiotic uptake of cyanobacteria that, over time, were integrated as organelles^1^. Primary plastids, direct descendants of this process, are found in three evolutionary lineages: red algae, green plants and glaucophytes^2^. Photosynthesis later spread horizontally through ‘secondary’ endosymbioses, resulting in most other eukaryotic algae^3^,^4^. These organisms are genetically complex and have been likened to ‘Matryoshka’ (Russian nesting dolls)^5^, with a cyanobacterium nested within a red alga that is further nested within a secondary host cell. As most genes that are retained from endosymbionts end up in the secondary host’s nuclear genome^6^, they present difficulties in molecular phylogenetic investigations. Although the secondary movement of green plastids appears well established^7^, the evolution of red-lineage plastids remains among the most controversial problems in broad-scale eukaryotic phylogenomics^8^,^9^,^10^.

Four major algal taxa contain plastids descended secondarily from red algal primary plastids, the Ochrophyta, Haptophyta, Cryptophyta and Dinophyta (dinoflagellates)^4^,^9^. Possible evolutionary or systematic connections among these groups have been suggested for over 60 years^11^. In 1981, Cavalier-Smith^12^ proposed the former three as members of a distinct kingdom, the Chromista, connecting their plastids through a single secondary endosymbiotic uptake of a red alga. This interpretation later was expanded as the chromalveolate hypothesis to include the Dinophyta, moving the proposed single endosymbiosis back to a common ancestor of chromists and the Alveolata, a group of protists that includes dinoflagellates, ciliates and apicomplexan parasites^13^.

Both alveolates and chromists include numerous heterotrophic forms that lack plastids altogether^14^. Ochrophytes, in particular, have long been recognized as related most closely to various heterotrophic organisms (for example, oomycetes) based on shared cytological features^15^,^16^; collectively they are grouped into the Heterokonta^12^ (or Stramenopiles^16^). A monophyletic Heterokonta also has been supported consistently by molecular phylogenetic analyses^17^,^18^,^19^, and cryptophytes and haptophytes fall outside this group based on all available evidence (both cytological and molecular). Therefore, if chromalveolate plastids are related by direct descent from a single endosymbiosis, they must have been lost from aplastidial heterokont and alveolate groups. More recently, molecular studies also have identified heterotrophic relatives of both cryptophytes and haptophytes^20^, although these relationships are more tentative because they are not, as yet, supported by the kinds of shared-derived cytological features that unite the Heterokonta.

Combined with evidence that chromist and dinoflagellate plastids all can be traced to a single red algal secondary endosymbiosis^5^,^8^,^21^, the increasing number of plastid losses implied by the chromalveolate model have prompted various proposals of multiple or serial endosymbioses^8^,^9^,^22^,^23^,^24^. To date, compelling evidence in support of a single, unambiguous evolutionary pathway has not emerged^8^, but it appears likely that the history of these plastids has included some level of reticulate rather than vertical inheritance. Additional endosymbioses would mean additional genetic layers in the genomes of these ‘Matryoshka algae’, further complicating their analyses through typical phylogenomic methods^25^. The problem is exacerbated by the generally weak tree-building signal in genomes for resolving deeper nodes of the eukaryotic tree of life^17^, as well as biases in sequence data that result in widespread phylogenetic artifacts^26^,^27^,^28^,^29^. Consequently, although phylogenomic investigations easily recover most classically defined taxa, and often cluster them into larger assemblies, they have difficulty producing consistent, strongly supported relationships among major eukaryotic lineages.

The relatively weak phylogenetic associations across major eukaryotic groups can allow other statistical patterns to emerge, which we have explored to assess impacts from past endosymbioses on chromist genomes. In an earlier study of heterokonts, we uncovered a strong relationship between the overall similarity of a given query genome to other eukaryotic groups, as measured by top matches in BLAST searches, and the number of sequences in the databases from each target group^30^. This relationship likely reflects the combined effects of expanded paralogous gene families and a reduced chance that ancestral genes and gene families have been lost from larger and/or multiple genomes (see ref. 30 for expanded discussion). Basically, the more potential matches present in a database, the greater the probability that a given query sequence will produce a match simply by chance. Later, we expanded those analyses to show that the relationship holds consistently across a diversity of eukaryotes for which complete genome data were available (Supplementary Fig. 1).

The stochastic properties of this relationship allow the use of regression analyses to generate null models for the expected similarity of any individual genome to those of other eukaryotic groups. Outliers, that is, taxa significantly more similar to a query than expected, can be identified from studentized residuals^31^. Such strong deviations rise above other sources of variation, for example, phylogenetic noise or individual horizontal gene transfers^32^, which are captured in the typical residual variance around the regression line. Therefore, they likely reflect either a close phylogenetic relationship or the impact of endosymbiotic gene transfer (EGT)^6^,^33^ between two more divergent taxa. If the deviation reflects a relatively recent divergence between nuclear genomes, then it is representative of consistent phylogenetic signal across the genome and the relationship is expected to be recovered using typical phylogenomic analyses^30^ (and see Supplementary Fig. 1). In the case of the three chromist algal groups it is more likely to reflect EGT, given that they have not been linked closely through phylogenetic analyses of nuclear genomes, but they clearly are connected by the common presence of red secondary plastids. Here, we present results that reject direct monophyletic inheritance of chromist plastids with plastid losses accounting for non-photosynthetic chromists and, further, provide strong evidence for a specific pathway of serial endosymbioses that accounts for the disjunct phylogenetic distribution of chromist plastids.

## Results

### Linear regressions and detection of outliers

**Figure 1:**
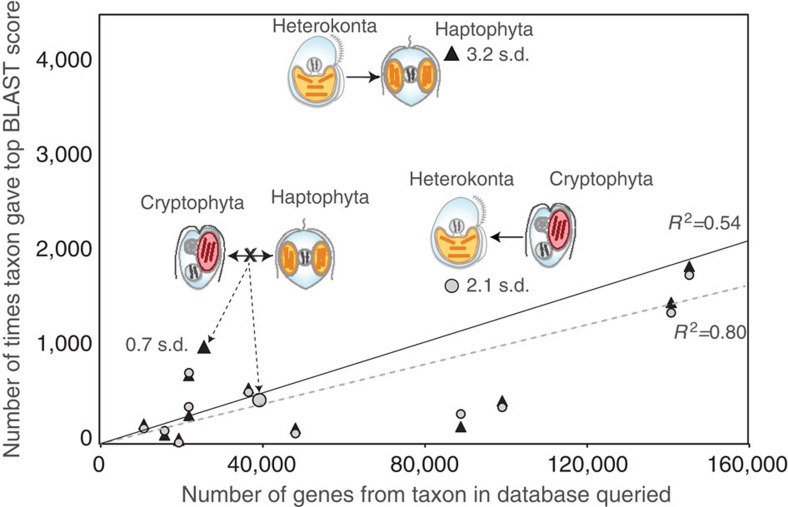
Evidence of EGT from regression analyses with significant outliers. Linear regressions on relationships between the number of most similar sequences (measured as top BLAST matches) from 13 other eukaryotic lineages to all inferred protein-encoding genes in haptophyte (solid line, black triangles) and cryptophyte (dashed line, grey circles) genomes. The largest outliers are highlighted with studentized residuals (residual divided by its standard deviation; ‘s.d.’ on figure) and images of the taxa that share more genes than expected based on the model, heterokonts in both cases. For comparison, the data points that indicate measures of similarity between cryptophytes and haptophytes genome also are highlighted. No studentized residual is provided for the data point indicating the number of top BLAST hits to haptophytes using the cryptophyte genome as query, because it falls at the predicted value from the regression line. Arrows indicate the proposed direction of EGT based on our overall model, except for the double-pointed arrow (the X indicating no evidence of EGT in either direction), which shows the reciprocal results between haptophytes and cryptophytes. A query’s matches to its own group (for example, haptophyte to haptophyte) are not counted, meaning these reciprocal data points are both unpaired on the figure.

We performed linear regressions and calculated studentized residuals for the number of most similar BLAST hits to all taxa using complete genomes from three of the four major chromalveolate algal lineages. Because no complete, well-annotated genome is available from dinoflagellates, we could not include them in our analyses. All regressions showed strong and significant relationships and revealed an interesting pattern with respect to outlier taxa. Although there appears to be no strong evidence to suggest a close relationship between host cells of haptophytes and heterokonts^19^, studentized residuals show that *Emiliania huxleyi* (Haptophyta) is significantly more similar to heterokonts (>3 s.d.) than predicted by the regression model (Fig. 1). An analysis using *Guillardia theta* (Cryptophyta) as the query genome produced similar results; in this case, the association with heterokonts was not significant, although at a level (>2 s.d.) that generally is considered to be potentially significant in analyses of studentized residuals, and worthy of further scrutiny (see ref. 31 for full discussion). In contrast, there was no indication that *Emiliania* and *Guillardia* genomes are more similar to each other than predicted by the regression model, regardless of which genome was used as the query (Fig. 1). This latter result is interesting because it is at odds with the widely discussed Hacrobia hypothesis, which argues that cryptophytes and haptophytes are the most closely related of the three chromist algal groups^34^. When a randomly chosen ochrophyte genome (the diatom *Phaeodactylum tricornutum*) was used as a reciprocal query, cryptophytes and haptophytes were the two largest outliers, although neither deviated significantly from their predicted values (Supplementary Fig. 2 and Supplementary Table 1). Thus, regressions provide strong support for an endosymbiotic association between heterokonts and haptophytes, suggest one between heterokonts and cryptophytes and give no indication that cryptophytes or haptophytes are related either phylogenetically or through a direct endosymbiosis.

**Figure 2:**
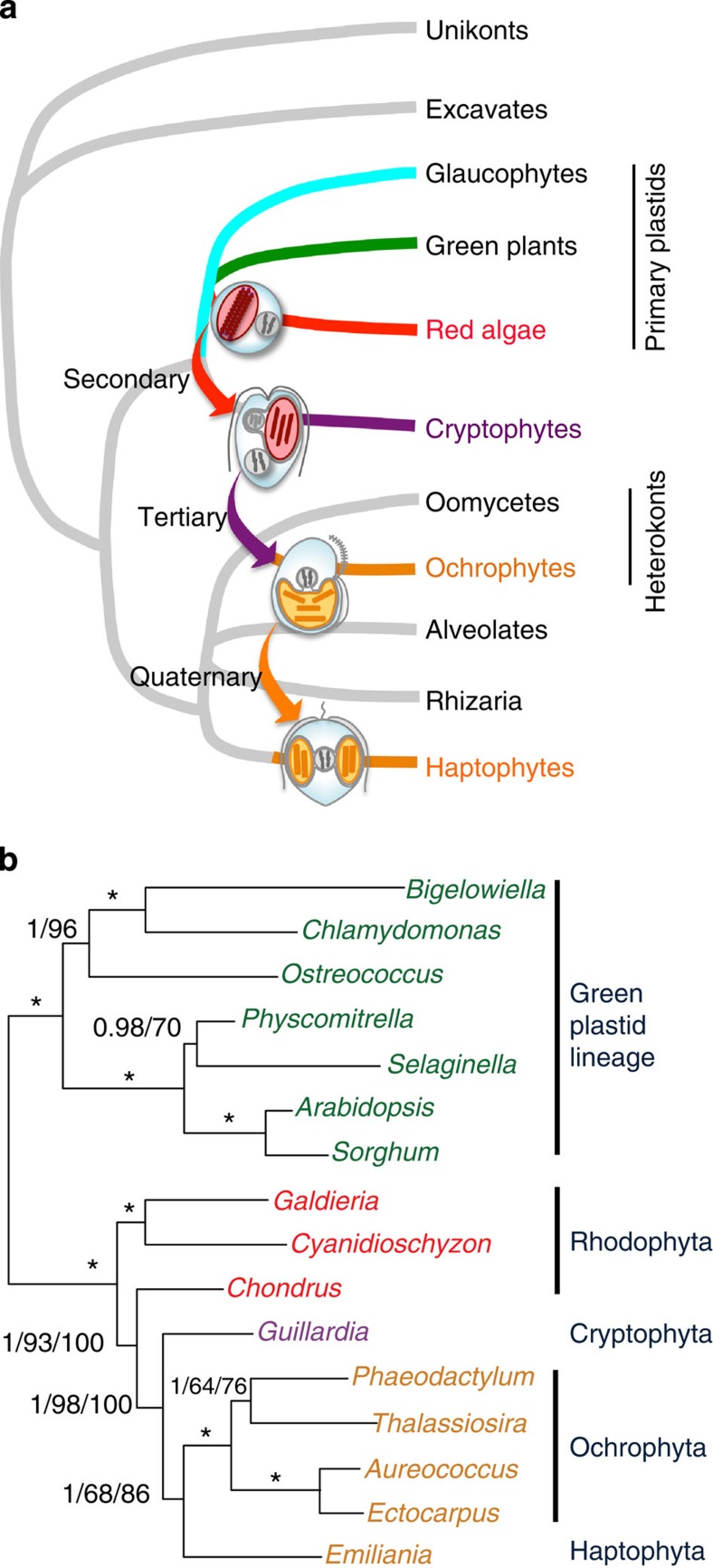
Model of serial plastid endosymbioses and a test using genes from plastid genomes. (**a**) Model of serial plastid endosymbioses suggested by regression analyses. The relationships depicted agree with general inferences from eukaryotic phylogenomics that heterokonts are not closely related to cryptophytes or haptophytes, and that each of the three groups emerge from mutually exclusive clades containing heterotrophic relatives. With respect to our proposed model of serial endosymbioses, the specific topology of the tree is not important, only that the three chromist algal groups do not form an exclusively monophyletic grouping that excludes aplastidial heterokonts. (**b**) Tree of plastid relationships based on an alignment of 5,818 amino-acid positions from genes inherited directly through the plastid genome, an independent data set for testing the model of plastid transfer inferred from EGT. The tree shown was recovered using both Bayesian and maximum-likelihood (ML) approaches. Bayesian posterior probabilities and ML bootstrap support values are provided for each node, with a star indicating 1.0 and 100% support, respectively. A second bootstrap value on nodes in the red plastid clade is from ML analyses performed in the absence of the green plastid lineage as outgroup. Bayesian probabilities are based on 5,000 sampled trees and bootstrap support values are from 1,000 replicates in each case.

The overall results of regression analyses suggest a pattern of serial endosymbioses, including the tertiary adoption of a cryptophyte by the ancestor of ochrophytes, and then a quaternary uptake of an ochrophyte into the ancestor of haptophytes. Figure 2a depicts these serial transfers mapped onto a tree of eukaryotic relationships adapted from the recent phylogenetic investigation of cryptophyte and haptophyte evolution by Burki *et al*.^19^ Because the regressions alone indicate only greater similarities than expected between query genomes to target taxa, they do not polarize plastid transfers in this direction; however, cryptophytes retain a residual red algal nucleus from the original secondary endosymbiosis^35^, which is absent from either of the other two groups. Thus, a series of endosymbioses beginning with haptophytes is unreasonable because it requires a highly unlikely scenario of retention and loss of the nucleomorph, or the even less likely assumption that the nucleomorph was regenerated in cryptophytes. A more recent quaternary endosymbiosis between haptophytes and ochrophytes also is consistent with strong similarities between the two in plastid pigmentation and ultrastructure, which often led to the inclusion of haptophytes within ochrophyte classes in historical classification systems (see ref. 36 for review).

### Contingency tests of model predictions

The model of plastid evolution that we propose makes explicit predictions as to where specific footprints of endosymbiosis (that is, significant evidence of EGT) should be found. First, if cryptophytes donated their plastid to establish ochrophytes, the transfer presumably occurred after the ochrophyte ancestor diverged from other heterokonts that remained heterotrophic. Thus, there should be evidence of EGT from cryptophytes into ochrophytes, but not into the heterotrophic relatives of ochrophytes. To test this prediction, we carried out Fisher’s exact tests on the numbers of most similar BLAST matches to four heterokonts, two photosynthetic and two heterotrophic, to determine whether evidence of EGT from cryptophytes was significantly greater than in control genomes with no putative genealogic or endosymbiotic relationship to either group. To avoid complications from shared photosynthetic pathways, which clearly make algal genomes more similar to each other under any circumstances, only genes presumably unrelated to plastid functions were included in these analyses. Both photosynthetic heterokonts tested, the diatom *Phaeodactylum* and pelagophyte *Aureococcus*, contained highly significant evidence of EGT compared with control genomes (Table 1). In contrast, no significant evidence was found for EGT from cryptophytes in the heterotrophic oomycete *Phytophthora* or protist *Blastocystis*. In fact, in three of four tests the non-photosynthetic heterokont showed a weaker putative impact from cryptophyte genes than did the paired control genome (Table 1). Thus, Fisher’s exact tests offer consistent and highly significant support for the hypothesized tertiary endosymbiosis between cryptophytes and ochrophytes.

We next tested predictions from the second hypothesized endosymbiosis, the quaternary adoption of an ochrophyte by the ancestor of haptophytes. In this case, because heterotrophic and photosynthetic heterokonts are closely related, most genes unrelated to plastid function should be relatively similar between the two. Consequently, evidence of EGT into haptophytes should be detectable from all heterokonts regardless of metabolic lifestyle. This prediction was strongly supported in all Fisher’s exact tests (Table 1). We note that if a plastid transfer occurred in the opposite direction, from a haptophyte to the ancestor of ochrophytes, then no significant association would be expected between non-photosynthetic heterokonts and haptophytes (see cryptophyte example above). Therefore, not only do results from contingency tests support specific predictions of the model inferred objectively from regression analyses, they also provide significant evidence against alternative hypotheses with mutually exclusive predictions (see further discussion below).

### Phylogenetic analyses of plastid genes

We performed one additional test of the proposed model of serial endosymbioses. Because genes retained within plastids have not moved between genomes, and were not included in our regressions and contingency tests, they represent independent data that should track the pathway of plastids through the respective chromalveolate lineages. Phylogenetic analyses using 5,818 inferred amino-acid positions from plastid genes were fully consistent with our proposed model (Fig. 2b). Cryptophyte plastids emerged from within red algae and as the sister group to an ochrophyte–haptophyte clade. Although this tree is not compelling evidence by itself, particularly given the small number of chromist plastid genomes included, it takes on greater significance as an *a posteriori* test of an explicitly defined phylogenetic hypothesis.

To avoid introducing additional variables, plastid genes were sampled only from those photosynthetic organisms included in our primary investigation of nuclear genomes. Nevertheless, our results agree with inferences from broader phylogenetic analyses carried out by Baurain *et al*.^18^ in their previous investigation of the chromalveolate model. Although neither study showed haptophyte plastids originating from within ochrophytes, as might be expected from the endosymbiotic transfer proposed, this could require much broader sampling of the global diversity of ochrophyte plastid genomes. Ochrophytes and haptophytes are believed to have emerged at around the same time during the late Proterozoic^37^, meaning the proposed plastid transfer must have originated from an early branch of ochrophytes, certainly before the broad radiation of most extant forms. Presumably, a substantial fraction of early diverging ochrophytes have been lost to the multiple mass extinctions that have punctuated more than 500 million years of intervening evolution^38^. Thus, it would be surprising if initial sampling had recovered haptophyte plastids nested within ochrophytes, rather than as their sister group. These factors also could explain why the horizontally transferred bacterial *rpl36* gene, which has been cited as a diagnostic feature linking cryptophyte and haptophyte plastids^39^, has not been found in ochrophyte plastid genomes to date.

## Discussion

For most of the last 15 years, the chromalveolate hypothesis has been the dominant model of red-lineage plastid evolution; it assumes a single secondary origin of plastids in the common ancestor of chromist algae, dinoflagellates and related heterotrophic taxa. Consequently, plastids must have been lost from the ancestors of chromalveolate groups where they are not currently present, including oomycetes and other aplastidial heterokonts. If the hypothesis of a continuous vertical descent of chromist plastids is correct, then what we have identified as a footprint of cryptophyte-EGT in ochrophyte genomes would reflect a phylogenetic rather than an endosymbiotic relationship. If so, that phylogenetic signal (as measured in genes unrelated to plastid function) also should be present in *Phytophthora* and *Blastocystis*, which share a more recent common ancestor with ochrophytes than do other chromist algae. Instead, our results (Table 1) provide significant evidence that the greater than expected similarity between cryptophyte and ochrophyte genomes does not predate the divergence between ochrophytes and aplastidial heterokonts. This is most reasonably explained by EGT from a tertiary cryptophyte endosymbiont, and is strong evidence against the vertical descent of chromist plastids from a single ancestral secondary endosymbiosis. As such, it is an explicit falsification of the core assumption of the chromalveolate hypothesis, and is in line with several other recent investigations that found evidence inconsistent with various aspects of that model^8^,^18^,^19^,^30^. It also emphasizes the importance of understanding how EGT has shaped photosynthetic eukaryotes, particularly ‘Matryoshka algae’ with complex, chimeric genomes.

Most previous inferences of EGT as evidence of a past endosymbiosis have been based on some indeterminate number of discrepant gene phylogenies suggesting the presence of foreign ‘algal’ genes in a given genome. Such trees can be interpreted as endosymbiotic footprints, but also could be examples of more general horizontal gene transfer or phylogenetic/sampling artifacts^32^,^40^. Our use of objective regression models and explicit negative controls in contingency tests shows that the footprints of EGT proposed in this study rise significantly above what can be expected from more random sources of variation within eukaryotic genomes. Nevertheless, even such highly significant support for greater than expected similarities among chromist algal genomes does not provide rigorous evidence for EGT on a gene-by-gene basis. This will require further investigations of our proposed model of serial endosymbioses, including careful and rigorous analyses of individual genes and gene sets that underlie the genome-wide signals that we uncovered.

Beyond supporting a specific pathway of endosymbiosis through chromist algae, our results also provide strong evidence against alternative models of higher-order red-lineage plastid evolution. For example, if all three, or even any two chromist algal lineages had been founded by independent secondary endosymbioses involving a red alga, we should not find significant endosymbiotic connections both between cryptophytes and ochrophytes, and between ochrophytes and haptophytes. Independent secondary endosymbioses predict that at least one of the chromist algae would show a significant endosymbiotic association with red algae only, and not with either of the other two chromist groups. We also note that, unlike independent secondary events, connecting chromist plastids through serial endosymbioses is consistent with evidence that all chromalveolate plastids trace back to a single secondary red algal endosymbiont^8^.

Likewise, an independent tertiary endosymbiosis between cryptophytes and haptophytes is inconsistent with the absence of an association between their genomes in regression analyses (Fig. 1). More importantly, it is contradicted by the highly significant support that we find for EGT from ochrophytes into haptophytes (Fig. 1, Table 1); this would not be present had haptophytes adopted a cryptophyte endosymbiont directly. The highly significant footprint of genes from non-photosynthetic heterokonts in the haptophyte genome also rules out the endosymbiotic movement of a haptophyte into ochrophytes, as noted above.

The specific pattern of serial endosymbioses that we propose also explains conflicting phylogenetic signals uncovered previously across the complete *Emiliania huxleyi* genome^41^. Although the dominant tree-building signal present recovered haptophytes as sister to the large SAR^42^ complex (stramenopiles (haptophytes), alveolates, rhizarians), ~30% of individual nuclear genes showed a specific phylogenetic affinity with heterokonts^41^. In contrast, no strong, concerted affinities were found for either cryptophyte or red algal genes. This is fully consistent with the quaternary endosymbiosis that we propose between ochrophytes and haptophytes, but is not easily reconciled with most alternative models of chromist plastid evolution. In conclusion, the results of our investigation represent a qualitative advance in understanding how the major chromist lineages acquired photosynthesis. In addition, they highlight the utility of new and alternative approaches for untangling the complex and reticulate nature of plastid evolution.

## Methods

### Database creation and sequence similarity searches

The general approach to database construction, BLAST searches and parsing of BLAST results followed those described previously^30^. Briefly, to investigate signal from EGT in genomes of chromists and related taxa, a sequence database was constructed including all inferred protein-encoding genes from the complete genomes of 50 organisms distributed across 14 well-established eukaryotic lineages (Supplementary Table 2). BLASTP searches were performed against this database using all inferred protein sequences as indicated from each specific query genome described in our results. Each sequence from a given query genome was used in only one search against the complete database. To identify homologues from the databases most reliably, a stringent *E*-value cut-off of 1e−20 was used in all searches (Supplementary Data 1). All sequences returned using a given query genome were retained in a data set in ranked order for use in computational pipelines described below. Only complete, well-annotated genomes were used for two primary reasons. First, our statistical treatments comparing potential signals from EGT across genomes are based on the total number of genes present, which cannot be determined accurately from transcriptome data alone. In addition, because investigations of EGT seek to measure the contribution of foreign genes present in the organisms assessed, it is essential that such genes can be placed in a genomic context, and are not simply present as mRNAs that could represent external contamination.

### Sorting pipelines

We wrote a custom JAVA program (Supplementary Data 2) to parse output files from BLAST searches. Relevant data, including all sequences producing significant alignments (≤e−20), their source genomes, group designation and *E*-values, were extracted and formatted along with the query sequences. We then created a database using MySQL to store these entries to facilitate multiple further computations, including various complicated SQL queries generated on the basis of the research questions arising from regression analyses. Information from BLAST output files for all sequences returned from each given query genome was stored in a specific, individual table.

The JAVA program that we developed used two inputs. The first was a category file, indicating to which of the 14 eukaryotic groups each of the 50 genomes belonged. The 14 groups were labelled with group ID numbers. The second input was the BLAST output file described above. The program generated output files in the following format:

Input sequence/similar sequence found/hit#/similar sequence/group ID/group name/genome/score/e-value, where ‘similar sequence’ indicates whether any sequence above our cut-off value was returned for the query sequence. ‘Hit#’ (hit-number) stands for the sequential rank number of a given sequence returned in a BLAST search. For the results reported here, we focused largely on best matches, after first screening out matches to members of the target group to which the query genome belonged, including all self-hits to the query genome itself. Output tables constructed in this format could be loaded directly to our database and sorted in whatever manner required for a given statistical test. We then constructed various SQL queries to conduct counting and parsing functions against the data file. As a screen for genes unrelated to photosynthesis, we included a command that the gene must be present in at least two genomes from the broadly sampled heterotrophic taxa metazoa and/or fungi.

### Statistical analyses

Linear regressions were carried out in SPSS (version 20) without a constant, given that a data set with zero genes must yield zero BLAST matches. For each of the individual query genomes described in our results, regressions were run on the number of BLASTP hits returned from each major eukaryotic group (Supplementary Table 2) versus the number of genes in each group’s database. Studentized residuals were calculated in each case to determine whether significant outliers were present.

Fisher’s exact tests were carried out using online software as described previously^15^ to test specific predictions of the model of serial plastid evolution that emerged from regression analyses. Two control genomes were chosen for each test from among those contained within our large database using the following criteria; (1) they were from organisms with no inferred history of photosynthesis, (2) they had no inferred phylogenetic or endosymbiotic associations with any of the algal lineages tested and (3) they were the closest in size (smaller and larger, respectively), among the genomes in our database, to the algal genome tested. Significance of one-tailed *P* values for multiple tests were determined using a sequential Bonferroni test^43^ with an *a priori ∝*=0.001.

### Phylogenetic analyses

A tree of plastid relationships was generated using inferred amino-acid sequences from the 11 largest genes retained in all plastid genomes from the 16 photosynthetic species included in our analyses of EGT in nuclear genomes. To avoid complications from additional experimental variables, we only analysed plastid genes from the photosynthetic organisms already present in our large database, so that results were directly comparable to nuclear gene analyses. Sequences were aligned using MUSCLE in MEGA 5.2.2 (ref. 44). To minimize potential phylogenetic artifacts, we took a conservative approach, removing all regions with inferred gaps even if they appeared to align well otherwise. Tests for the most appropriate model of sequence evolution were performed for all individual alignments in MEGA 5.2.2, with a cpREV+G+I model inferred in all cases. A single concatenated alignment of 5,818 positions was created and used for tree reconstruction. Trees were inferred using maximum-likelihood in PhyML^45^ with 1,000 bootstrap repetitions, and by Bayesian inference using MrBayes^46^ run for 1 million generations with trees sampled every 100 generations. On the basis of standard deviations among split frequencies, the final 5,000 trees sampled were used to construct a Bayesian consensus tree and infer posterior probabilities. To determine effects (potential long-branch artifacts) from rooting the tree with the green plastid clade, we repeated the phylogenetic analyses (as described above) with a sub-alignment containing sequences only from red algal plastids and their chromist descendants.

## Author contributions

J.W.S. was involved in the overall study design and data interpretation, and was the primary author of the paper; J.S. performed statistical tests and phylogenetic analyses; J.Y. assembled the data sets and carried out BLAST analyses; H.G. wrote and implemented computational programs for parsing BLAST results; Q.D. was involved in design of the overall study and computational programs; J.H. participated in the overall study design and bioinformatic analyses. All the authors contributed editorially to the paper.

## Additional information

**How to cite this article:** Stiller, J. W. *et al*. The evolution of photosynthesis in chromist algae through serial endosymbioses. *Nat. Commun.* 5:5764 doi: 10.1038/ncomms6764 (2014).

## Supplementary Material

Supplementary Information Supplementary Figures 1-2, Supplementary Tables 1-2, and Supplementary References

Supplementary Data 1 Tables of top Blast matches among chromist groups used in the regressions depicted in Figure 1 and Supplementary Figure 2, and highlighted in Supplementary Table 1. These lists contain sequences responsible for the greater than expected similarities among chromist nuclear genomes; however, individual sequences should not be considered specific evidence of endosymbiotic gene transfer without explicit gene-by-gene analyses.

Supplementary Data 2 Java program files, along with explanatory readme file, contain scripts used to extract Blast matches used in statistical analyses. The Java program parses Blast output files, extracts the data needed and stores them in a database, so that various queries can be performed to assemble data for statistical analyses.

## Figures and Tables

**Table 1:** Fisher’s exact tests for significant impacts of EGT from non-photosynthetic genes in heterokont and haptophyte genomes.

**Query**		**Top BLAST search matches to:**		**Ratio (adjusted to genome size) target:control**	***P***
**Heterokonts**		**Cryptophyte**	**Control**		
*Phaeodactylum*	Photosynthetic	276	48*	3.55	1.7e−20
			185†	2.13	3.4e−16
*Aureococcus*	Photosynthetic	298	59*	3.13	2.1e−19
			225†	1.9	1.7e−13
*Phytophthora*	Non-photosynthetic	248	103*	1.49	0.0003
			554†	0.62	0.999
*Blastocystis*	Non-photosynthetic	75	50*	0.93	0.695
			255†	0.44	0.999
**Haptophyte**		**Heterokonts**			
*Emiliania*	Photosynthetic	1,088‡	436§	5.0	2.7e−204
			402†	2.0	8.3e−36
*Emiliania*	Photosynthetic	658||	456§	3.85	1.8e−110
			396†	1.62	1.9e−15

^*^Heterolobosea.

^†^Amoebozoa.

^‡^Matches to photosynthetic heterokonts.

^§^Fungi.

^||^Matches to non-photosynthetic heterokonts.

## References

[b1] Martin W. & Kowallik K. V. Annotated English translation of Mereschkowsky’s 1905 paper ’Uber natur und ursprung der chromatophoren im pflanzenreiche. Eur. J. Phycol. 34, 287–295 (1999).

[b2] Delwiche C. F. & Palmer J. D. inOrigins of Algae and their Plastids 11, ed. Bhattacharya D. 53–86Springer (1997).

[b3] Keeling P. J. The number, speed, and impact of plastid endosymbioses in eukaryotic evolution. Annu. Rev. Plant Biol. 64, 583–607 (2013). 23451781 10.1146/annurev-arplant-050312-120144

[b4] Gould S. B., Waller R. R. & McFadden G. I. Plastid evolution. Annu. Rev. Plant Biol. 59, 491–517 (2008). 18315522 10.1146/annurev.arplant.59.032607.092915

[b5] Petersen J., Teich R., Brinkmann H. & Cerff R. A. ‘green’ phosphoribulokinase in complex algae with red plastids: evidence for a single secondary endosymbiosis leading to haptophytes, cryptophytes, heterokonts, and dinoflagellates. J. Mol. Evol. 62, 143–157 (2006). 16474987 10.1007/s00239-004-0305-3

[b6] Timmis J. N., Ayliffe M. A., Huang C. Y. & Martin W. Endosymbiotic gene transfer: organelle genomes forge eukaryotic chromosomes. Nat. Rev. Genet. 5, 123–135 (2004). 14735123 10.1038/nrg1271

[b7] Rogers M. B., Gilson P. R., Su V., McFadden G. I. & Keeling P. J. The complete chloroplast genome of the chlorarachniophyte *Bigelowiella natans*: Evidence for independent origins of chlorarachniophyte and euglenid secondary endosymbionts. Mol. Biol. Evol. 24, 54–62 (2007). 16990439 10.1093/molbev/msl129

[b8] Petersen J. . *Chromera velia*, endosymbioses and the rhodoplex hypothesis—plastid evolution in cryptophytes, alveolates, stramenopiles, and haptophytes (CASH lineages). Genome Biol. Evol. 6, 666–684 (2014). 24572015 10.1093/gbe/evu043 PMC3971594

[b9] Sanchez-Puerta M. V. & Delwiche C. F. A hypothesis for plastid evolution in chromalveolates. J. Phycol. 44, 1097–1107 (2008). 10.1111/j.1529-8817.2008.00559.x 27041706

[b10] Bodyl A. Do plastid-related characters support the chromalveolate hypothesis? J. Phycol. 41, 712–719 (2005).

[b11] Chadefaud M. Les cellules nageuses des algues dans l’embranchement des Chromophycées. *Comptes Rendus Hebd*. Acad. Sci. Paris 231, 788–790 (1950).

[b12] Cavalier-Smith T. Eukaryote kingdoms: seven or nine? Biosystems 14, 461–481 (1981). 7337818 10.1016/0303-2647(81)90050-2

[b13] Cavalier-Smith T. Principles of protein and lipid targeting in secondary symbiogenesis: euglenoid, dinoflagellate, and sporozoan plastid origins and the eukaryote family tree. J. Eukaryot. Microbiol. 46, 347–366 (1999). 18092388 10.1111/j.1550-7408.1999.tb04614.x

[b14] Adl S. M. . The revised classification of eukaryotes. J. Eukaryot. Microbiol. 59, 429–514 (2012). 23020233 10.1111/j.1550-7408.2012.00644.x PMC3483872

[b15] Sparrow F. K. Aquatic Phycomycetes University Michigan Press (1960).

[b16] Patterson D. J. inThe Chromophyte Algae, Problems and Perspectives eds Green B. J., Leadbeater B. S. C., Diver W. L. 357–379Clarendon (1989).

[b17] Yoon H. S. . Broadly sampled multigene trees of eukaryotes. BMC Evol. Biol. 8, 14 (2008). 18205932 10.1186/1471-2148-8-14 PMC2249577

[b18] Baurain D. . Phylogenomic evidence for separate acquisition of plastids in cryptophytes, haptophytes and stramenopiles. Mol. Biol. Evol. 27, 1698–1709 (2010). 20194427 10.1093/molbev/msq059

[b19] Burki F., Okamoto N., Pombert J. F. & Keeling P. J. The evolutionary history of haptophytes and cryptophytes: phylogenomic evidence for separate origins. Proc. Biol. Sci. 279, 2246–2254 (2012). 22298847 10.1098/rspb.2011.2301 PMC3321700

[b20] Burki F. . Large-scale phylogenomic analyses reveal that two enigmatic protist lineages, Telonemia and Centroheliozoa, are related to photosynthetic chromalveolates. Genome Biol. Evol. 1, 231–238 (2009). 20333193 10.1093/gbe/evp022 PMC2817417

[b21] Stork S., Lau J., Moog D. & Maier U. G. Three old and one new: protein import into red algal-derived plastids surrounded by four membranes. Protoplasma 250, 1013–1023 (2013). 23612938 10.1007/s00709-013-0498-7

[b22] Bodyl A., Stiller J. W. & Mackiewicz P. Chromalveolate plastids: direct descent or multiple endosymbioses? Trends Ecol. Evol. 24, 119–121 (2009). 19200617 10.1016/j.tree.2008.11.003

[b23] Bodyl A. & Moszczynski K. Did the peridinin plastid evolve through tertiary endosymbiosis? A hypothesis. Eur. J. Phycol. 41, 435–448 (2006).

[b24] Teich R., Zauner S., Baurain D., Brinkmann H. & Petersen J. Origin and distribution of Calvin cycle fructose and sedoheptulose bisphosphatases in Plantae and complex algae: a single secondary origin of complex red plastids and subsequent propagation via tertiary endosymbioses. Protist 158, 263–276 (2007). 17368985 10.1016/j.protis.2006.12.004

[b25] Lane C. E. & Archibald J. M. The eukaryotic tree of life: endosymbiosis takes its TOL. Trends Ecol. Evol. 23, 268–275 (2008). 18378040 10.1016/j.tree.2008.02.004

[b26] Penny D., McComish B. J., Charleston M. A. & Hendy M. D. Mathematical elegance with biochemical realism: the covarion model of molecular evolution. J. Mol. Evol. 53, 711–723 (2001). 11677631 10.1007/s002390010258

[b27] Lockhart P. . Heterotachy and tree building: a case study with plastids and eubacteria. Mol. Biol. Evol. 23, 40–45 (2006). 16151191 10.1093/molbev/msj005

[b28] Shalchian-Tabrizi K. . Heterotachy processes in rhodophyte-derived secondhand plastid genes: implications for addressing the origin and evolution of dinoflagellate plastids. Mol. Biol. Evol. 23, 1504–1515 (2006). 16699169 10.1093/molbev/msl011

[b29] Philippe H., Delsuc F., Brinkmann H. & Lartillot N. Phylogenomics. Annu. Rev. Ecol. Evol. Syst. 36, 541–562 (2005).

[b30] Stiller J. W., Huang J. L., Ding Q., Tian J. & Goodwillie C. Are algal genes in nonphotosynthetic protists evidence of historical plastid endosymbioses? BMC Genomics 10, 484 (2009). 19843329 10.1186/1471-2164-10-484 PMC2770532

[b31] Cook R. D. & Weisberg S. Residuals and Influence in Regression Chapman and Hall (1982).

[b32] Stiller J. W. Experimental design and statistical rigor in phylogenomics of horizontal and endosymbiotic gene transfer. BMC Evol. Biol. 11, 259 (2011). 21923904 10.1186/1471-2148-11-259 PMC3190393

[b33] Martin W., Brinkmann H., Savonna C. & Cerff R. Evidence for a chimeric nature of nuclear genomes: eubacterial origin of eukaryotic glyceraldehyde-3-phosphate dehydrogenase genes. Proc. Natl Acad. Sci. USA 90, 8692–8696 (1993). 8378350 10.1073/pnas.90.18.8692 PMC47424

[b34] Okamoto N., Chantangsi C., Horák A., Leander B. S. & Keeling P. J. Molecular phylogeny and description of the novel Katablepharid *Roombia truncata* gen. et sp. nov., and establishment of the Hacrobia taxon nov. PLoS ONE 4, e7080 (2009). 19759916 10.1371/journal.pone.0007080 PMC2741603

[b35] Archibald J. M. Nucleomorph genomes: structure, function, origin and evolution. Bioessays 29, 392–402 (2007). 17373660 10.1002/bies.20551

[b36] Medlin L., Kooistra W. C. F., Potter D., Saunders G. & Andersen R. inOrigins of Algae and their Plastids 11, ed. Bhattacharya D. 187–219Springer (1997).

[b37] Parfrey L. W., Lahr D. J. G., Knoll A. H. & Katz L. A. Estimating the timing of early eukaryotic diversification with multigene molecular clocks. Proc. Natl Acad. Sci. USA 108, 13624–13629 (2011). 21810989 10.1073/pnas.1110633108 PMC3158185

[b38] Raup D. M. & Sepkoski J. J. Mass extinctions in the marine fossil record. Science 215, 1501–1503 (1982). 17788674 10.1126/science.215.4539.1501

[b39] Rice D. W. & Palmer J. D. An exceptional horizontal gene transfer in plastids: gene replacement by a distant bacterial paralog and evidence that haptophyte and cryptophyte plastids are sisters. BMC Biol. 4, 31 (2006). 16956407 10.1186/1741-7007-4-31 PMC1570145

[b40] Deschamps P. & Moreira D. Reevaluating the green contribution to diatom genomes. Genome Biol. Evol. 4, 683–688 (2012). 22684208 10.1093/gbe/evs053 PMC5635612

[b41] Read B. A. . Pan genome of the phytoplankton *Emiliania* underpins its global distribution. Nature 499, 209–213 (2013). 23760476 10.1038/nature12221

[b42] Burki F. . Phylogenomics reshuffles the eukaryotic supergroups. PLoS ONE 2, e790 (2007). 17726520 10.1371/journal.pone.0000790 PMC1949142

[b43] Rice W. R. Analyzing tables of statistical tests. Evolution 43, 223–225 (1989). 10.1111/j.1558-5646.1989.tb04220.x 28568501

[b44] Tamura K. . MEGA5: molecular evolutionary genetics analysis using maximum likelihood, evolutionary distance, and maximum parsimony methods. Mol. Biol. Evol. 28, 2731–2739 (2011). 21546353 10.1093/molbev/msr121 PMC3203626

[b45] Guindon S. . New algorithms and methods to estimate maximum-likelihood phylogenies: assessing the performance of PhyML 3.0. Syst. Biol. 59, 307–321 (2010). 20525638 10.1093/sysbio/syq010

[b46] Ronquist F. . MrBayes 3.2: efficient Bayesian phylogenetic inference and model choice across a large model space. Syst. Biol. 61, 539–542 (2012). 22357727 10.1093/sysbio/sys029 PMC3329765

