# Supplementary material for: The evolution of photosynthesis in chromist algae through serial endosymbioses
Source: Nat Commun. 2014 Dec 10;5:5764. doi: 10.1038/ncomms6764 (PMC4284659; doi:10.1038/ncomms6764)
Supplement: Supplementary Information — Supplementary Figures 1-2, Supplementary Tables 1-2, and Supplementary References [file ncomms6764-s1.pdf]

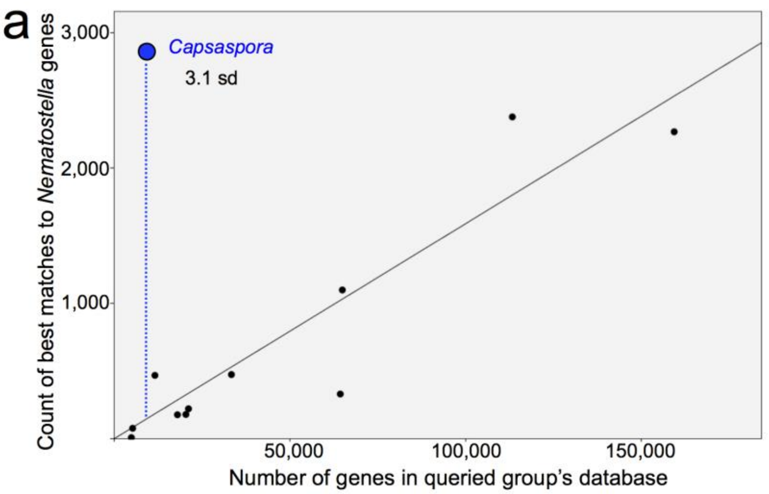

b

Query genome	R^2
Cyanidioschyzon (Rhodophyta)	0.72
Emiliania (Haptophyta)	0.54
Guillardia (Cryptophyta)	0.80
***Nematostella* (Metazoa)**	**0.59**
Nematostella (w/o *Capsaspora*)	0.90
Paramecium (Ciliata)	0.91
Phytophthora (Heterokonta)	0.91
Plasmodium (Apicomplexa)	0.90
Selaginella (Viridiplantae)	0.86
Thalassiosira (Heterokonta)	0.75
Yarrowia (Fungi)	0.88

Supplementary Figure 1. Examples and summary of strong relationships between genome similarity and database size. (**a)** Example of the linear regressions run on representatives of 14 major eukaryotic lineages, using group database size (in number of protein-encoding genes) as the independent variable, and the number of top matches to the query genome as the dependent variable. The plot shown is for *Nematostella vectensis*, chosen because it is average-sized among metazoan genomes. The relationship is significant (P = 0.002) with a relatively high coefficient of determination (R^2 = 0.59); however, *Capsaspora owczarzaki*, recognized as the closest protistan relative to metazoans + choanoflagellates[1], is identified as a significant positive outlier (studentized residual > 3) and substantially reduces the model's goodness-of-fit. When *Capsaspora* is removed from the analysis, the model fit increases substantially (R^2 = 0.92). This example demonstrates how regressions can be used to model the predicted similarity of a given genome to those from diverse taxa, and to identify lineages that are significantly more similar than expected. In this case, the similarity is due to an established genealogical relationship, which is recovered strongly in phylogenetic analyses. In the absence of such a clear relationship, significant regression outliers can result from EGT as indicted by the lower R^2 value for the regression analysis of the haptophyte, *Emiliania huxleyi*, which is fully described in Fig. 1 of the main text. (**b)** Summary of coefficients of determination for comparable regressions performed on BLAST results from representatives of diverse eukaryotic taxa. All regressions were highly significant at P < 0.001 and with high R^2 values, except for the *Nematostella* example highlighted above, with *Capsospora* included as a target taxon (P = 0.002). Coefficients of determination tended to be lower for genomes of all photosynthetic organisms (shown in red), likely reflecting general impacts of shared genes from EGT among all photosynthetic groups present.

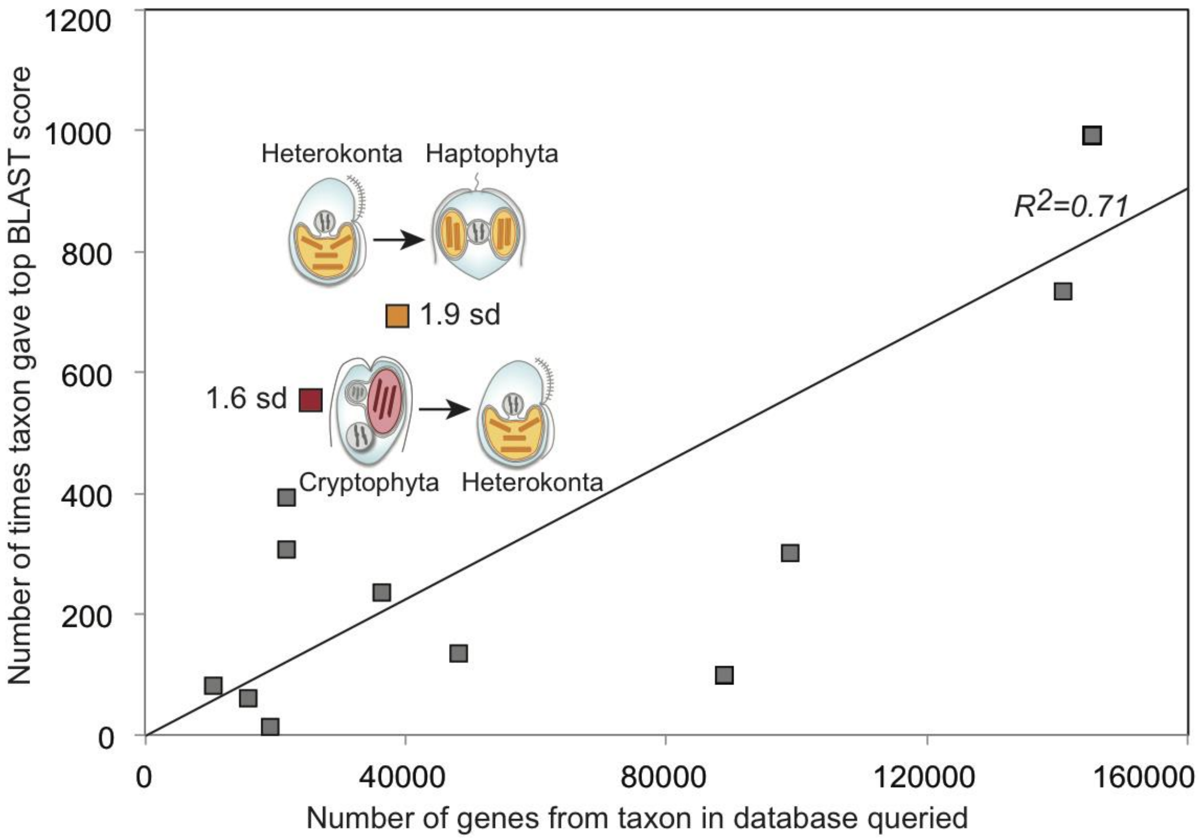

Supplementary Figure 2. Linear regressions on relationships between the number of most similar sequences (measured as top BLAST matches) from 13 other eukaryotic lineages to all inferred protein encoding genes in the ochrophyte (*Phaeodactylum tricornutum*) genome. The largest outliers are highlighted by colored data points, studentized residuals (s.d.), and an image for the direction of gene transfer predicted by our evolutionary model of serial endosymbioses. Although the cryptophyte and haptophyte genomes are the two largest outliers from values predicted by the regression model, in neither case is the studentized residual significant. This result is consistent with our model that ochrophytes have an endosymbiotic history with both of the other chromist algae, meaning signals from genes indicating an endosymbiotic association are predicted to be split between haptophytes and cryptophytes.

Supplementary Table 1. Number of top matches and studentized residuals from BLAST searches using haptophyte (*Emiliania*), cryptophyte (*Guillardia*) and ochrophyte (*Phaeodactylum*) genomes as queries. SRE = studentized residual error. Yellow shaded boxes indicate results that suggest a directional model of endosymbioses, which are highlighted in regressions shown in Fig. 1 and Supplemental Fig. 2. Heterokonts show a greater than expected similarity to both haptophytes and cryptophytes when the latter two are used as individual queries. This suggests an endosymbiotic association of heterokonts with both taxa. The reciprocal results, using an ochrophyte as the query genome, effectively splits the signal from EGT between the cryptophyte and haptophyte target genomes. In contrast, there is no indication of EGT between cryptophytes and haptophytes when either genome is used as the BLAST query.

**Target group**	**Database size**	**Query genome**					
		Emiliania		***Guillardia***		***Phaeodactylum***	
		**Top hits**	**SRE**	**Top hits**	**SRE**	**Top hits**	**SRE**
Viridiplantae	145189	1861	-0.1	1778	0.9	993	1.0
Metazoa + *Capsospora*	140718	1482	-0.5	1383	-0.1	733	-0.2
Fungi	99042	451	-1.0	411	-1.6	302	-1.0
Ciliata	88824	180	-1.1	322	-1.5	98	-1.6
Heterokonta	86590	4093	3.2	1656	2.1		
Apicomplexa + *Perkinsus*	48025	164	-0.5	136	-0.9	136	-0.5
Haptophyta	39125			468	0.2	689	1.9
Amoebozoa	36451	583	0.1	560	0.5	237	0.2
Cryptophyta	25472	1011	0.7			557	1.6
Rhodophyta	21770	297	0.0	735	1.3	307	0.7
Rhizaria	21708	716	0.4	397	0.4	393	1.1
Euglenozoa	19253	38	-0.2	22	-0.4	12	-0.4
Heterolobosea	15753	93	-0.1	137	-0.1	60	-0.1
Apusozoa	10627	207	0.1	188	0.2	83	0.1

Supplementary table 2. Organisms included in this investigation, grouped into major eukaryotic lineages targeted in BLAST searches. Sizes of protein-encoding data sets are shown for each complete genome, along with total numbers of sequences present for each of the major lineages.

Major taxa and species	Genome size	Source for data
**Amoebozoa (3)**		
Entamoeba histolytica	8,163	http://www.ncbi.nlm.nih.gov/
Dictyostelium discoideum	13,315	http://www.ncbi.nlm.nih.gov/
Acanthamoeba castellanii	14,974	http://www.ncbi.nlm.nih.gov/
Total:	36,452	
**Apicomplexans plus *Perkinsus* (5)**		
Toxoplasma gondii	8,103	http://toxodb.org/
Plasmodium falciparum	5,337	http://www.ncbi.nlm.nih.gov/
Neospora caninum	7,122	http://toxodb.org/
Cryptosporidium parvum	3,805	http://cryptodb.org/
Perkinsus marinus	23,658	http://www.ncbi.nlm.nih.gov/
Total:	48,025	
**Apusozoa**		
Thecamonas trahens	10,627	http://www.broadinstitute.org/
**Ciliates (3)**		
Paramecium tetraurelia	39,521	http://paramecium.cgm.cnrs-gif.fr/
Tetrahymena thermophila	24,725	http://ciliate.org/index.php/
Sterkiella histriomuscorum	24,578	http://www.ncbi.nlm.nih.gov/
Total:	88,824	
**Cryptophyta**		
Guillardia theta (genome + nucleomorph)	25,504	http://genome.jgi.doe.gov/
**Euglenozoa (2)**		
Leishmania major	8,406	http://tritrypdb.org/
Trypanosoma cruzi	10,847	http://www.ncbi.nlm.nih.gov/
Total:	19,253	
**Fungi and relatives (9)**		
Magnaporthe grisea	11,054	http://www.broadinstitute.org/
Neurospora crassa	9,907	http://www.broadinstitute.org/
Aspergillus fumigatus	9,888	http://www.broadinstitute.org/
Ustilago maydis	6,522	http://www.broadinstitute.org/
Coprinopsis cinerea	13,342	http://www.broadinstitute.org/
Rhizopus oryzae	17,459	http://www.broadinstitute.org/
Antonospora locustae	2,606	http://forest.mbl.edu/
Allomyces macrogynus	19,446	http://www.broadinstitute.org/
Batrachochytrium dendrobatidis	8,818	http://www.broadinstitute.org/
Total:	99,042	
**Haptophyte**		
Emiliania huxleyi	39,125	http://genome.jgi-psf.org/
**Heterokonta (7)**		
Phaeodactylum tricornutum	10,025	http://genome.jgi-psf.org/
Thalassiosira pseudonana	11,390	http://genome.jgi-psf.org/
Ectocarpus siliculosus	16,589	http://www.ncbi.nlm.nih.gov/
Aureococcus anophagefferens	11,501	http://genome.jgi-psf.org/
Blastocystis hominis	6,020	http://www.ncbi.nlm.nih.gov/
Phytophthora infestans	15,743	ftp://ftp.ensemblgenomes.org/
Pythium ultimum	15,322	ftp://ftp.ensemblgenomes.org/
Total:	86,590	
**Heterolobosea**		
Naegleria gruberi	15,753	http://genome.jgi-psf.org/
**Metazoa + protist relatives (7)**		
Homo sapiens	33,615	http://www.ncbi.nlm.nih.gov/
Drosophila melanogaster	19,789	Metazome v3.0
Caenorhabditis elegans	25,816	http://www.ncbi.nlm.nih.gov/
Nematostella vectensis	24,780	http://www.ncbi.nlm.nih.gov/
Monosiga brevicollis	9,196	http://genome.jgi-psf.org/
Capsaspora owczarzaki	8,792	http://www.broadinstitute.org/
Sphaeroforma arctica	18,730	http://www.broadinstitute.org/
Total:	140,718	
**Rhizaria**		
Bigelowiella natans	21,708	http://genome.jgi-psf.org/
**Rhodophyta (3)**		
Cyanidioschyzon merolae	5,016	http://merolae.biol.s.u-tokyo.ac.jp/
Chondrus crispus	9,580	http://www.ncbi.nlm.nih.gov/
Galdieria sulphuraria	7,174	http://www.ncbi.nlm.nih.gov/
Total:	21,770	
**Viridiplantae (6)**		
Arabidopsis thaliana	35,378	http://www.ncbi.nlm.nih.gov/
Sorghum bicolor	29448	Phytozome v9.0
Physcomitrella patens	35940	http://www.ncbi.nlm.nih.gov/
Selaginella moellendorffii	22,285	Phytozome v9.0
Chlamydomonas reinhardtii	14,413	http://www.ncbi.nlm.nih.gov/
Ostreococcus tauri	7,725	http://genome.jgi-psf.org/
Total:	145,189	

Supplementary References

1. Suga, H. *et al.* The *Capsaspora* genome reveals a complex unicellular prehistory of animals. *Nat. Commun.* **4,** 2325 (2013).
